# Controllable Physical Synergized Triboelectricity, Shape Memory, Self‐Healing, and Optical Sensing with Rollable Form Factor by Zn cluster

**DOI:** 10.1002/advs.202200441

**Published:** 2022-04-22

**Authors:** Dahye Ahn, Jingzhe Sun, Seunghye Han, Jiwoo Lee, Songah Jeong, Seokjun Cha, Seonmyeong Noh, Hyeongsub Choi, Bingqi Ren, Hyeonseok Yoon, Hyungwoo Kim, Jong‐Jin Park

**Affiliations:** ^1^ Department of Polymer Science and Engineering Chonnam National University Gwangju 61186 Republic of Korea

**Keywords:** anti‐counterfeit tag, pore structure, rollable self‐healing touchpad, shape memory rotating triboelectric nanogenerator, Zn cluster

## Abstract

To build devices offering users comfortable experience, it is important to focus on form factor and multifunctionality. In this study, for the first time, multifunctional Zn clusters with shape memory, self‐healing, triboelectricity, and optical sensing synergized with rollable form factor are designed and fabricated by coordinating —COO^−^ and Zn^2+^. As pore forming agent, Zn clusters produce hierarchical porous structure depending on Zn amount. Zn clusters are applied as message transmitters and charge containers in optical sensing and corona charge injection, respectively. Moreover, Zn clusters in PVB‐COO‐Zn serve as positive tribomaterial due to Zn ion doping effect, increasing the output performance as the Zn amount reaches 20 wt%. In addition, injecting positive charge into PVB‐COO‐Zn 20 lead to more than 24 times increase in output performance compared to those of non‐porous structures. The reversibility of Zn clusters endows shape memory and self‐healing, synergized with the rollable form factor. The rollability is implemented using the long alkyl chain and the energy absorption of porous structure, providing damage resistance. The advancements in this work provide opportunities for multifunctional and unique applications (shape memory rotating‐triboelectric nanogenerator, rollable self‐healing touchpad, hidden tag) synergized with rollability that accomplishes working in broadened condition in near future.

## Graphical abstract

Multifunctions with triboelectricity, shape memory, self‐healing, and optical transmitting property integrated with rollable form factor controlled by Zn clusters are designed and fabricated through coordinating carboxylate (—COO^−^) and Zn ion (Zn^2+^). Enhanced rollability is endowed by introducing long alkyl chain and energy absorption of porous structure, which dissipates energy.

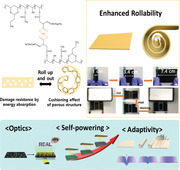

## 1 Introduction

At present, new material solutions are constantly being developed to cope with the increasing demand for more efficient and convenient devices. To offer a device user a comfortable experience, it is crucial to develop materials that have multifunctional applications, which include flexibility, self‐sustainability, and adaptivity.^[^ ^1^, ^2^, ^3^, ^4^, ^5^, ^6^, ^7^ ^]^ As an aspect of self‐sustainability, triboelectricity has recently attracted substantial because it solves the problem of energy depletion by converting the wasted mechanical energy into electrical energy through physical contact of two different materials.^[^ ^8^, ^9^, ^10^ ^]^ The fact that repeated friction can damage a material surface necessitates the adaptive property that allows the shape or function of a material to return to its original state through shape memory or self‐healing.^[^ ^11^, ^12^, ^13^, ^14^ ^]^ However, most of the existing studies to this point have dealt with conventional structures and functions of such applications, which hinders the development of efficient and unique technology. One potential way to solve this problem is to integrate functions with transformable configurations (form factor).^[^ ^15^ ^]^

Form factor including aspects such as foldability, stretchability, wearability, and rollability has become a critical part of creating a convenient environment for device users. Among the aspects of form factor, rollability is particularly attractive due to its high portability with extendibility and flexibility under extreme daily conditions.^[^ ^16^, ^17^, ^18^, ^19^ ^]^ Prior studies have used paper,^[^ ^20^, ^21^ ^]^ silicon rubber,^[^ ^22^ ^]^ and soft polymeric material^[^ ^23^, ^24^, ^25^, ^26^ ^]^ to realize a rollable form factor. However, rollability has still been restricted in these studies due to the poor durability and flexibility of the materials when being rolled up and out. It is therefore necessary to develop soft and durable materials through modifications that allow them that can better endure the mechanical force from repeated rolling up and out than the materials designed in previous studies.

The chemical or physical crosslinking method in polymer is a widely used method for preparing mechanically durable materials. Since chemical crosslinks are formed through covalent interactions that have strong and non‐reversible bonds, the use of physical crosslinks formed by weak and reversible bonds is highly recommended for applications requiring toughness for repetitive rolling.^[^ ^27^, ^28^, ^29^ ^]^ One of the actively used physically crosslinking methods is metal coordination, wherein crosslinks can be formed through non‐covalent interactions via ligands surrounding metal ions. The currently preferred method is the coordination of metal ion and anion from polymer due to its spontaneous formation and the stable bond.^[^ ^30^, ^31^ ^]^ Moreover, due to the presence of metal ions and organic ligands, various functional properties such as dielectrics, luminescence, magnetism, catalysis, stimuli‐responsiveness, and shape‐memory behavior have been demonstrated,^[^ ^32^, ^33^, ^34^, ^35^ ^]^ but there is still a lack of research integrating a rollable form factor and multifunctions, which restricts the utilization of devices in various conditions.

**Figure 1:**
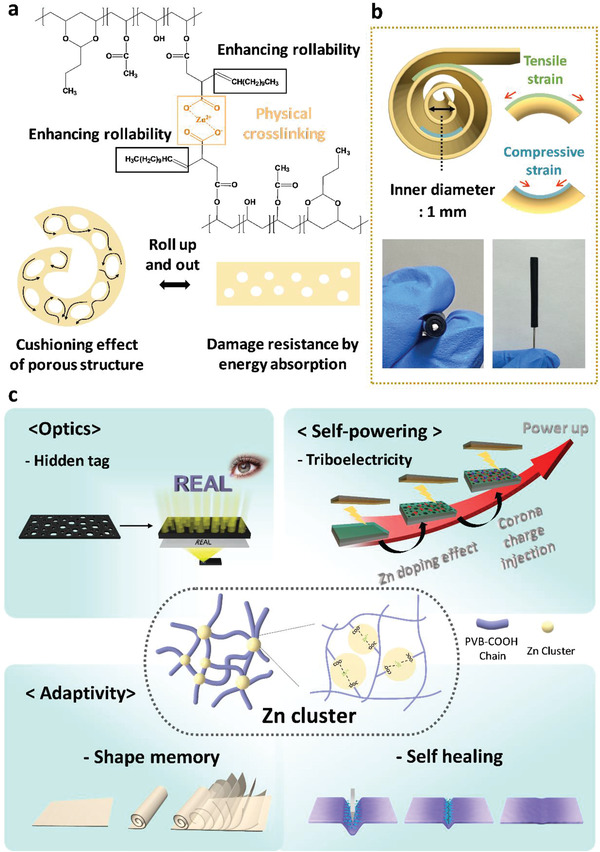
a) Chemical structure of the formation of PVB‐COO‐Zn through the Zn‐carboxylate bond and schematic illustration of the cushioning effect of the morphological structure. b) Schematic illustration of the rollability of PVB‐COO‐Zn film denoted in terms of the inner diameter and photographic images of PVB‐COO‐Zn rolled around a metal wire (inner diameter: 1 mm) while maintaining its shape. c) Schematic illustration of the multifunctional Zn cluster that can be applied to fields involving optics, self‐powering, and adaptivity.

In this study, Zn clusters with the multifunctions of triboelectricity, shape memory, self‐healing, and optical transmitting property integrated with a rollable form factor were designed and fabricated for the first time by coordinating carboxylate (—COO^−^) and Zn ion (Zn^2+^) (Table S1, Supporting Information). For rollability, poly(vinyl butyral) (PVB), a random terpolymer composed of acetal, acetate, and hydroxyl groups, was chosen for its strong binding ability and adhesiveness derived from the acetal group.^[^ ^36^, ^37^ ^]^ To prepare the film for repetitive rolling up and spreading out, dodecenylsuccinic anhydride (DDSA) and long alkyl chain moiety were introduced in the PVB side chain to provide flexibility, thus resulting in a ring opening reaction. Long alkyl side chains produce more free volume than short side chains, thus leading to an internal plasticizing effect.^[^ ^38^ ^]^ Moreover, with the cushioning effect of the porous structure derived from Zn clusters, energy from both tensile and compressive stress was absorbed, thus making the film resistant to damage when subjected to repetitive rolling up and out (**Figure** **1**a). The introduction of long alkyl chains side by side between polymer chains and physical crosslinking of —COO^−^ and Zn^2+^ had the effect of enhancing the rollability of the film, thus allowing it to be rolled up around a support with an inner diameter of ≈1 mm. For a material to tolerate being continuously rolled up and out, it must be able to withstand the tensile and compressive stress generated by the outer and inner axis, respectively (Figure 1b).^[^ ^39^ ^]^ After the PVB modification, Zn clusters were formed by coordinating —COO^−^ and Zn^2+^; these can implement multifunctions depending on the Zn amount. The Zn clusters hierarchically generated a porous structure, and the number and the size of the pores both varied depending on the Zn amount. This porous structure could be used as an optical pathway for a hidden tag to control the amount of light intensity through controlling the number and size, and that it could offer damage resistance to absorb energy during rolling. Zn clusters can be used as the positive tribomaterial in TENG, as the Zn clusters possess a positive ion and work as ion dopant. The output performance of TENG was increased with the Zn amount, and it could be further enhanced through corona charge injection with the contribution from the porous structures. Furthermore, the Zn clusters served as physical crosslinking points that were thermally reversible and that rendered temporary shapes, thus enabling shape memory and self‐healing of the surface damage for multiple applications based on the rollable form factor (Figure 1c). In this work, diverse functions with transformable geometries that could respond to various circumstances were achieved by controlling the amount of Zn clusters, thus expanding the applicability of such materials for the fields of optics, triboelectric energy, and adaptivity.

## 2 Results and Discussions

**Figure 2:**
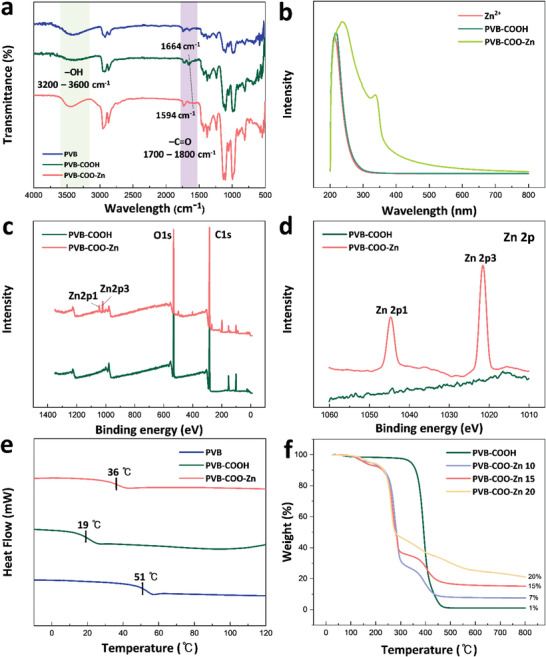
a) FT‐IR graphs of PVB, PVB‐COOH, and PVB‐COO‐Zn. b) UV–vis spectra of Zn^2+^, PVB‐COOH, and PVB‐COO‐Zn. c) XPS graphs of PVB‐COOH and PVB‐COO‐Zn. d) XPS spectra of Zn 2p XPS spectrum of PVB‐COOH and PVB‐COO‐Zn. e) DSC graphs of PVB, PVB‐COOH, and PVB‐COO‐Zn. f) TGA graphs of PVB, PVB‐COOH, and PVB‐COO‐Zn 10,15, and 20.

As depicted in Figure 1, PVB modification was performed to make Zn clusters. Through the reaction between DDSA and the hydroxyl group (—OH) in PVB, a carboxylic acid group (—COOH) was introduced in the PVB side chain by the ring opening reaction (PVB‐COOH) (Figure S1, Supporting Information). After introducing anhydride with a long alkyl side chain, the glass transition temperature (*T*_g_) was dropped below room temperature to impart rollability in the room temperature condition. Comparing *T*_g_ with anhydride having a shorter alkyl side chain, it was confirmed that the use of DDSA was more suitable for achieving rollability (Figure S2, Supporting Information). Different molar ratios of —OH in PVB to DDSA were used for the modification of PVB. Molar ratio 1:4 was the most suitable amount considering the *T*_g_ for rollability and triboelectricity depending on the ring opening result (Table S2, Supporting Information). A similar characterization was conducted using ^1^H nuclear magnetic resonance (NMR) spectra (Figures S3 and 4, Supporting Information). By reacting PVB‐COOH with ZnCl_2_, PVB‐COO‐Zn was achieved through the ionic interaction between —COO^−^ and Zn^2+^, and this acted as a Zn cluster. The PVB‐COO‐Zn samples were named “PVB‐COO‐Zn *n*,” where *n* represents the weight percentage. PVB‐COOH and PVB–COO–Zn were characterized through Fourier transform infrared spectroscopy (FT‐IR) (**Figure** **2**a). After PVB was modified, the peak at 3200 to 3600 cm^−1^ corresponding to the —OH bond decreased as a result of the participation of —OH in the ring opening reaction. The peak intensity around 1664 cm^−1^ increased as a result of the increased —C═O bond due to the introduction of DDSA. In addition, the peak shift from 1664 to 1594 cm^–1^ indicates the coordination effect of Zn^2+^ and —C═O, thus revealing that the Zn clusters were formed in PVB‐COO‐Zn.^[^ ^40^ ^]^ The peak that corresponded to —COO^−^ was observed at 1570 cm^−1^ (Figure S5, Supporting Information).^[^ ^41^ ^]^ Furthermore, the formation of PVB‐COO‐Zn was confirmed through UV–vis spectrum and X‐ray photoelectron spectroscopy (XPS). As shown in Figure 2b, neither the Zn^2+^ solution nor the PVB‐COOH solution showed an absorbance peak in the range of 300–350 nm, whereas the PVB‐COO‐Zn^2+^ solution showed a peak at 334 nm, thus verifying the successful formation of the coordination bond of Zn carboxylate (Figure S6, Supporting Information).^[^ ^42^ ^]^ The chemical compositions of the surfaces were further confirmed through XPS. The survey spectra shown in Figure 2c indicate the presence of Zn, O, and C elements. Figure 2d shows the high‐resolution spectra of the Zn 2p photoelectron lines of PVB‐COOH and PVB‐COO‐Zn. The spin–orbit transitions of the Zn 2p1 and 2p3 binding energy peaks, respectively, appear at ≈1044 and 1021 eV,^[^ ^43^ ^]^ which is not confirmed in PVB‐COOH. The thermal properties of PVB‐COOH and PVB‐COO‐Zn were confirmed through differential scanning calorimeter (DSC) analysis and thermogravimetric analysis (TGA) (Figure 2e,f). Due to the plasticizing effect of long alkyl side chains, *T*_g_ decreased from 51 to 19 °C after the modification of PVB. Following the physical crosslinking, *T*_g_ increased from 19 to 40 °C. As the Zn amount increases, the physical crosslinking point increases, and *T*_g_ increases in turn (Figure S7, Supporting Information). In addition, the thermal stability of PVB‐COO‐Zn was revealed through TGA. The residual weights of PVB‐COO‐Zn at 800 °C remained at 7%, 15%, and 20% for PVB‐COO‐Zn 10, 15, and 20, respectively, which were ≈7 to 20 times more than the residual weight of PVB‐COOH. This may be ascribed to the metal ion incorporated in the polymer chain through physical crosslinking. To examine the thermomechanical behavior of PVB‐COO‐Zn, dynamic mechanical analysis (DMA) was conducted via tension mode. The result showed that PVB‐COO‐Zn 15 had a modulus of 300 MPa at room temperature. It also showed a peak near 40 °C, which corresponds to the *T*_g_ of PVB‐COO‐Zn 15 (Figure S8, Supporting Information). The interaction between PVB‐COO^−^ and Zn was performed through density functional theory (DFT) calculations, and the binding of the PVB‐COO^−^ with Zn was confirmed to be thermodynamically favorable for the physical crosslinking distance of 2.04−2.06 Å (Figure S9 and Table S3, Supporting Information).

**Figure 3:**
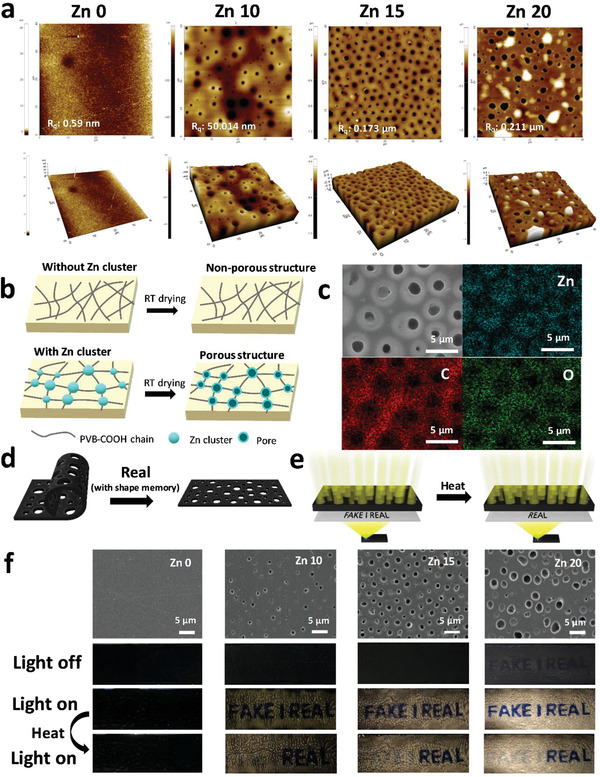
a) AFM images of porous structures in PVB‐COO‐Zn according to Zn amount. b) Schematic illustration of mechanism of pore formation depending on the existence of Zn clusters. c) SEM image and element mapping images of Zn, C, and O elements of porous structures in PVB‐COO‐Zn. Double checking of system using PVB‐COO‐Zn‐CB film for anti‐counterfeit: d) Primary checking through shape recovery of PVB‐COO‐Zn‐CB film. e) Secondary checking through hidden tag and its mechanism. f) SEM images and photographic images of hidden tag fabricated by PVB‐COOH‐CB and PVB‐COO‐Zn‐CB films according to Zn amount and results under light and heat.

To demonstrate the properties that distinguish it from conventional materials, PVB‐COO‐Zn was morphologically analyzed. **Figure** **3** shows the porous structure depending on the Zn amount as observed by atomic force microscopy (AFM) and scanning electron microscopy (SEM), the pore forming mechanism, and its applications. As the Zn amount increases, the pores become larger in size and increase in number, which is accompanied by increases in the number and sizes of the Zn clusters. It was confirmed that the pores were mainly distributed in 600–800, 1000–1200, and 1200–1400 nm in Zn 10, Zn 15, and Zn 20, respectively (Figure S10, Supporting Information). Related to the growing number and size of pores, the surface roughness values (*R*_q_) increased gradually from 0.59 nm to 0.211 µm (Figure 3a). The generation of porous structures can be explained by the existence of Zn clusters. When Zn clusters are absent, PVB‐COOH chains are well dispersed in organic solvent, thus causing no difference in surface tension and eventually forming a non‐porous structure after evaporation. By contrast, in PVB‐COO‐Zn, the Zn clusters have no affinity with organic solvent, thus causing an organic solvent to be condensed near Zn.^[^ ^44^ ^]^ After evaporation, pore structures are produced in PVB‐COO‐Zn (Figure 3b). These were further confirmed through energy‐dispersive X‐ray spectroscopy (EDS) elemental mapping. Zn was mainly distributed in the pore structures, unlike C and O, which were distributed where pores were not observed. This result confirmed that Zn clusters affected the sizes of the porous structures depending on the amount of Zn (Figure 3c). The formation of pores can be explained using Equation (1).

(1) $$E\;=\;\Gamma 2\pi r_{0}\;-\;\sigma \pi r_{0}^{2}$$

where *E* represents the net energy of pore formation, Γ represents edge energy, and *σ* represents surface tension.^[^ ^45^ ^]^ The pore formation can be explained by the edge energy and tension energy, which are respectively related to the expansion of the pore perimeter and the removal of an area.^[^ ^46^, ^47^ ^]^ When there is no Zn cluster, there is no crosslinked point, and as a result the net energy for creating pores does not exist due to the absences of an expansive driving force and the difference in surface tension needed for the removal of an area. By contrast, when there are Zn clusters, the edge energy needed for the creation of the boundary of pores (Γ2*πr*_0_) increases as the pore size increases, which is proportional to the pore edge. In addition, being affected by the surface tension difference from the crosslinking point (Zn clusters), the tension energy ($\sigma \pi r_{0}^{2}$) is reduced with the elimination of an area, which is supported by the decrease in the contact angle as shown in Figure S11, Supporting Information. As the edge energy increases and the tension energy decreases, there is an increase in the total net energy needed for pore formation, thus leading to the formation of a porous structure. The created porous structure was used in hidden tag to control the optical pathway for an anti‐counterfeit application with shape memory. A hidden tag was fabricated to allow for determinations of authenticity through a double check system with shape recovery and light transmittance (Figure 3d,e). First, by examining the shape recovery of the hiding layer through the shape memory effect, the authenticity can be designated as real. Second, the visibility of characters (FAKE | REAL) through light transmission depending on pore numbers and sizes were utilized for the anti‐counterfeit tag. Considering that the characters are initially hidden before becoming clearly visible with the application of heat, the Zn 15 condition (mean pore size: 0.724 µm) was considered to be optimal for anti‐counterfeit tags (Figure 3f and Video S1, Supporting Information). As a result, the pore structures are determined to be controllable for the use of optical pathways as message transmitters.

**Figure 4:**
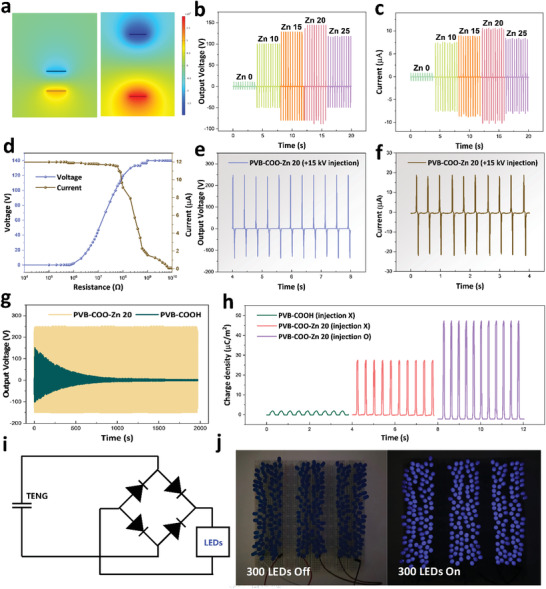
a) Working mechanism of PVB‐COO‐Zn‐TENG simulated by COMSOL. b) Output voltage of PVB‐COO‐Zn‐TENG according to Zn amount. c) Output current of PVB‐COO‐Zn‐TENG according to Zn amount. d) Output voltage and current according to load resistance. e) Output voltage of PVB‐COO‐Zn 20‐TENG after injection. f) Output current of PVB‐COO‐Zn 20‐TENG after injection. g) Stability test results of PVB‐COOH‐TENG and PVB‐COO‐Zn 20‐TENG after injection. h) Surface charge density of PVB‐COOH, PVB‐COO‐Zn 20 without injection and PVB‐COO‐Zn 20 with injection. i) Circuit illustration for lighting up LEDs. j) Photographic images of 300 LEDs before and after lighting.

The created pore structure was also used in charge trapping site to enhance triboelectricity following corona charge injection. Zn clusters were used as polymeric tribomaterial that retained the self‐sustaining properties and advantage of enhancing output performance through ion doping and corona charge injection. Since ZnCl_2_ has a net positive charge, PVB‐COO‐Zn was used as a positive friction material.^[^ ^48^ ^]^ Taking PTFE as a negative material, the working mechanism in contact‐separation mode is simulated by COMSOL and is depicted elsewhere (**Figure** **4**a and Figure S12, Supporting Information). Once the PVB‐COO‐Zn film comes in contact with the PTFE film, negative and positive triboelectric charges are gained by PTFE and PVB‐COO‐Zn, respectively, due to their differing abilities to lose or gain electrons. When two friction materials separate, the potential difference between PTFE and PVB‐COO‐Zn gradually increases, thus causing electrons to move through the external circuit to balance the potential difference; the flow of electrons continues until the two friction materials are fully separated. When the PVB‐COO‐Zn film approaches the PTFE film, electrons drive between the electrodes in a reverse direction, thus returning to the initial state.^[^ ^49^ ^]^ Figure 4b, c shows the output voltage and output current based on the Zn amount. As the Zn amount increases, the output performances were enhanced from the ion doping effect of Zn clusters, and the best performance was found in PVB‐COO‐Zn 20 (144 V, 10.6 µA). However, the output performance decreased from PVB‐COO‐Zn 25 due to the decrease in the contact area resulting from the increased pore size in the same area (4 × 4 cm), which hinders triboelectricity due to the decreasing friction area.^[^ ^50^ ^]^ The output voltage, output current, and output power density were measured under varying load resistance (Figure 4d). With the increase in the load resistance, the output voltage increased and the output current decreased. At a resistance of 10^8^ Ω, the output power density was at its maximum (746 mW m^−2^) (Figure S13, Supporting Information). Moreover, the output performance can be further enhanced by corona charge injection under porous structures to increase the surface charge density (Figure 4e,f). To optimize the charge injection condition, different voltages of charges were injected, and 15 kV was found to be the most suitable (Figure S14, Supporting Information). 15 kV of positive ions was injected onto the PVB‐COO‐Zn 20 film (mean pore size: 0.857 µm) that showed the best performance before injection. Following this positive ion injection, the output voltage increased ≈24 times from 10 to 248 V compared to that of the PVB‐COOH sample. The output current also increased from 0.83 to 18 µA with the increase in output voltage. When the pore size of the Zn cluster is below 0.5 µm, the output performance was only limited to 140 V with a low increasing ratio, even though high corona charge injection voltage was applied to the film. When the pore size of the Zn cluster is above 0.7 µm, the output performance increases to 248 V at the same corona charge injection of 15 kV. This can be attributed to the high increase in the charge trap effect due to the maximized amount of Zn positioned in the pore size. As a result, in corona charge injection, the pore structures effectively served as charge containers to increase the triboelectricity. However, when different voltages of a negative charge were injected, the output performance gradually decreased. This ensured a reduction in the potential difference of PTFE and PVB‐COO‐Zn, thus verifying that PVB‐COO‐Zn was conducive for positive material in triboelectric series (Figure S15, Supporting Information). A stability test was also conducted to examine the output voltage after charge injection. When corona charge was injected into PVB‐COOH, the output voltage decreased to 10 V. However, when it was injected into PVB‐COO‐Zn 20, the output voltage remained unchanged for 2000 s, thus demonstrating that the porous structure of PVB‐COO‐Zn worked as a charge trapping site that increased the charge retention time (Figure 4g and Figure S16, Supporting Information). The Zn doping effect and the corona charge injection, which was possible due to the Zn clusters, enhanced the surface charge density (Figure 4h). With this increased output performance and charge retention time, 300 LEDs were lit up with the electrical energy harvested by PVB‐COO‐Zn‐TENG of the contact‐separation mode (Figure 4j and Video S2, Supporting Information). Figure 4i shows a circuit illustration of PVB‐COO‐Zn‐TENG.

**Figure 5:**
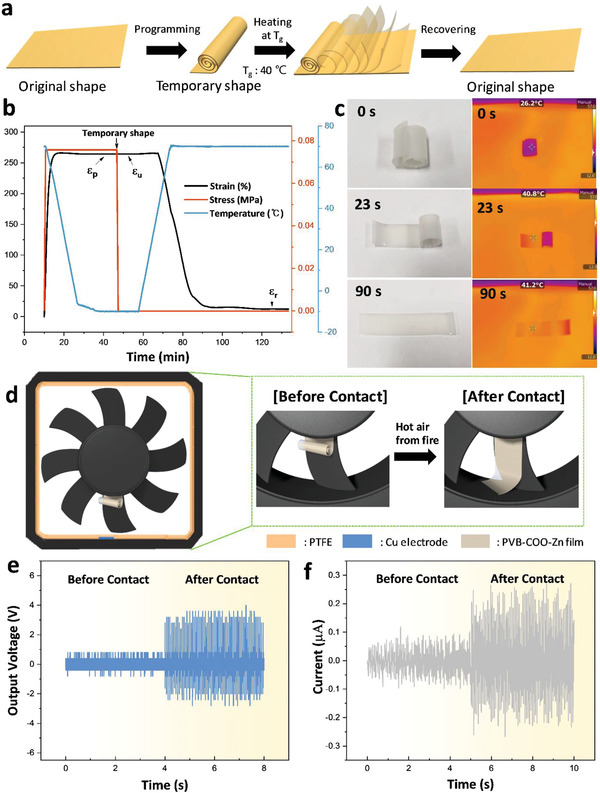
a) Schematic illustration of shape memory working mechanism of PVB‐COO‐Zn 15. b) Shape memory property of PVB‐COO‐Zn 15 as measured by DMA in force‐controlled mode. c) Photographic images (left) and IR camera images (right) taken at 0, 23, and 90 s. d) Schematic illustration of SMR‐TENG before and after the film contacts the blade. e) Output voltage of SMR‐TENG before and after the film contacts the blade. f) Output current of SMR‐TENG before and after the film contacts the blade.

The created Zn cluster was designed to endow the adaptive property with repetitive rollability. Adaptive properties like shape memory and self‐healing were also observed under the Zn cluster condition, since Zn clusters serve as a physical crosslinking point. **Figure** **5**a shows the overall process of shape memory. To program the shape of the PVB‐COO‐Zn film, the film was rolled by heating the polymer above *T*_g_ to bring it to a flexible state. The rolled shape could be fixed by cooling the film below *T*_g_, where the mobility of the polymer chains was restricted. Next, heat was applied to the rolled film. The vigorous mobility of the polymer chains led the film to return to its original state.^[^ ^51^ ^]^ The shape memory properties of PVB‐COO‐Zn were characterized through cyclic thermomechanical testing using DMA under controlled force mode (Figure 5b). The experiment results showed that the PVB‐COO‐Zn 15 film had shape fixity (*R*_f_) and shape recovery capabilities (*R*_r_) of 100% and 95%, respectively, thus indicating that PVB‐COO‐Zn has good shape memory behavior (*ε*_*u*_ = *ε*_*p*_ = 265%, *ε*_*r*_ = 12%).^[^ ^52^ ^]^ The 3D test record is shown in Figure S17, Supporting Information. Based on the thermomechanical property, the film can be fixed in the rolled state or the folded state for the shape memory test, as shown in Figure 5c. At the initial state, the film was rolled in the process of shape memory programming. After placing the sample and setting the heating plate temperature to 40 °C, the sample gradually recovered to its original flat form after 90 s as the temperature reached 40 °C (Figure 5d and Video S3, Supporting Information). To confirm that the shape memory effect existed in various forms, shape memory in the box type was observed, as it recovered to the original unfolded form (Figure S18, Supporting Information). As we already described the merit of the rollable form factor, the rollable form can return its volume to its normal status through programming by shape memory. In emergency situations, the rollable form can fully extend to a flat form to represent an alert of a dangerous condition. We integrated the shape recovery feature with triboelectric property in a 2D form by fabricating the shape memory rotating‐TENG (SMR‐TENG) as a fire alarm sensor for fire monitoring in mountains. Figure 5d shows the schematic illustration of the SMR‐TENG and the status before and after film contacts the blade. Before a fire breaks out, the film attached to the wind blade is in the rolled‐up shape since there is no thermal source to activate the shape memory to recover to its original flat shape. When the film is in this rolled‐up shape, the friction area is small, resulting in low output performance due to the reduced contact area. After a fire breaks out, the rolled film changes its shape to the flat state in response to the hot air from the fire. At the same time, the sliding contact area of the film and the PTFE increases, which eventually improves the output performance. Figure 5e,f shows the output performance of SMR‐TENG as a result of the shape memory recovery. At the time, a dryer was used to generate heat wind over 40 °C. Before applying the heat wind, the film maintained its rolled‐up shape and rotated without sliding contact, thus causing no change in output performance. After applying heat wind under the speed of 8.2 m s^−1^ and temperature over 40 °C, the film was flattened by the shape memory effect (Figure S19, Supporting Information). This resulted in enhanced output performance due to the increased sliding contact area. Based on the difference in the output performance, SMR‐TENG exhibits great potential for practical applications as a fire alarm sensor.

**Figure 6:**
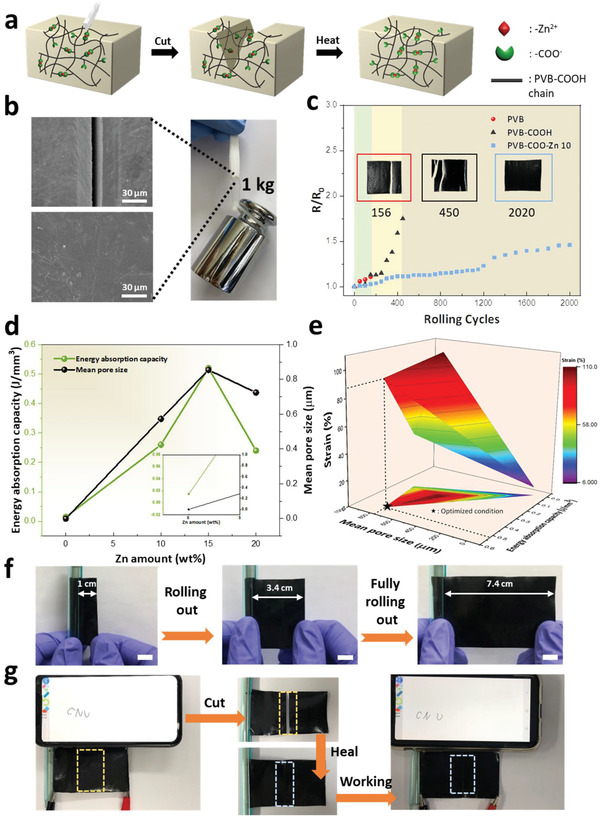
a) Schematic illustration of self‐healing mechanism. b) Self‐healed film sustaining a weight of 1 kg and SEM images of film surface before and after healing. c) Stability test of rollability and comparison between PVB, PVB‐COOH, and PVB‐COO‐Zn 10. d) Energy absorption capacity and mean pore size according to Zn amount. e) 3D graph with strain, mean pore size, and energy absorption capacity. f) Rollable self‐healing touchpad according to the degree of extension (scale bar: 1 cm). g) Working image of rollable self‐healing touchpad using original film and self‐healed film.

To improve the SMR‐TENG performance in response to surface scratches during rotation, self‐healing properties were introduced into PVB‐COO‐Zn as illustrated in **Figure** **6**a.^[^ ^53^, ^54^, ^55^ ^]^ When the film is deeply scratched, ionic bonds constituting Zn clusters are disconnected. After being heated over *T*_g_, polymer chains consisting of Zn clusters lose attraction to each other, leading them to move freely and causing the polymer chains on both sides of the scratched surface to get closer to each other.^[^ ^56^ ^]^ Finally, the ionic bonds of —COO^−^ and Zn^2+^ reassociate and form Zn clusters during cooling after reaching the healed status of the film along the Zn cluster.^[^ ^57^ ^]^ The stress–strain curves of PVB‐COO‐Zn 10 were demonstrated under varying healing times and temperatures. The film samples were cut in half with a razor blade before being later brought back into contact and healed. The original sample showed a strength of 0.45 MPa and a breaking strain of 110%. As the healing time increased, the healing efficiency increased as well, reaching up to 96% when healed for 1.5 h. Moreover, increasing the healing temperature was effective due to the high movement of Zn clusters resulting from —COO^−^ and Zn^2+^ and the increased inducement of reorientation. When the sample was healed at 60 °C for 1.5 h, the breaking strain and strength were recovered almost completely when compared to the original sample (Figure S20, Supporting Information). Figure 6b shows the healed surface of the deeply scratched sample as observed by SEM. Based on the optimized healing condition, the sample was healed almost perfectly to hold a weight of 1 kg. To confirm the self‐healing effect in composite, a touch pad based on 15 wt% of carbon black electrode was fabricated. The touch pad combined with rollability works by sensing electrical signals produced by hand writing. Here, considering the efficient movement of Zn clusters in self‐healing, PVB‐COO‐Zn 10 was found to be the best Zn amount condition for the preparation of a rollable self‐healing touchpad. With the contributions of the long alkyl side chain and the physical crosslinking between —COO^−^ and Zn^2+^, enhanced rollability was achieved in PVB‐COO‐Zn. The stability of PVB‐COO‐Zn 10 under repetitive rolling both up and out was tested and compared with the corresponding stabilities of PVB and PVB‐COOH (Figure 6c). The PVB electrode film began to exhibit a crack at 156 cycles; by contrast, the PVB‐COOH electrode film was stable until 450 cycles, indicating roughly three times more stability. This indicated that the long alkyl side chain accompanied by the plasticized effect and free volumes was conducive for repetitive rollability. When the Zn cluster was introduced in the PVB‐COOH chain, the PVB‐COO‐Zn 10 electrode film was durable for 2000 cycles, showing 13 times better stability than PVB electrode film. This revealed that the ionic interaction between Zn^2+^ and —COO^−^ had a positive effect on stress relaxation in repetitive rolling up and out, as confirmed by stress‐relaxation tests (Figure S21, Supporting Information). Among the samples, PVB‐COOH showed the highest residual stress ratio, indicating that the sample relaxed the stress slowly. By contrast, the PVB‐COO‐Zn composites showed considerable stress relaxation, meaning that the rebuilding of Zn‐carboxylate bond allowed for the rearrangement of the network.^[^ ^58^ ^]^ The rolling stability of PVB‐COO‐Zn after healing is shown in Figure S22, Supporting Information. Further, the porous structure had a considerable effect on rollability because of the fact that, to a certain extent, energy absorption capacity increases with increasing pore size.^[^ ^59^ ^]^ Figure 6d shows the mean pore size and energy absorption capacity depending on the Zn amount. Here, the energy absorption capacity can be obtained using the area of the stress–strain curve (Figure S23, Supporting Information).^[^ ^60^ ^]^ Energy absorption capacity, *E*_v_, is equal to the area under the stress–strain curve, which is expressed as

(2) $$E_{v}=\int_{0}^{\varepsilon_{\max}}\sigma d\varepsilon$$

where *σ* represents stress and *ε*_max_ represents maximum strain.^[^ ^61^ ^]^ In proportion to the Zn amount, the mean pore size increased from 0.573 to 0.857 µm. The energy absorption capacity was also increased with increasing pore size; specifically, the energy absorption capacity grew to 0.52 J mm^−3^ at Zn 15 wt%, which is 34 times larger than that of a non‐porous structure (0.015 J mm^−3^). However, the energy absorption capacity from Zn 20 wt% decreased to 0.24 J mm^−3^, thus resulting in reduced rollability. This can be interpreted to mean that when the pore size is over 0.857 µm, an increase in stiffness diminishes the energy absorption effect of the pores. Unlike the non‐porous structure, the porous structure with an empty space of a hierarchical distribution will be able to dissipate the energy in the manner of a cushioning effect, as illustrated in Figure 1a, while undergoing tensile and compressive strain.^[^ ^62^ ^]^ The physical property of the film also affects repetitive rollability, which is related to the strain.^[^ ^63^ ^]^ Figure 6e shows the correlation between strain, pore size, and energy absorption capacity. As strain increases, energy absorption increases, but at the point where strain is the highest, energy absorption is low. Since the physical property and pore formation are both related to Zn clusters, this indicates that the strain and the pore size should both be accounted for when considering energy absorption capacity. At the optimized point (marked with a star), the stress that can affect the form factor is dissipated through energy absorption. The dissipation of stress has damage resistance that prevents the film from being deformed by lowering the direct transfer of the tensile and compressive stresses, which are generated during repeated rolling to the form factor.^[^ ^64^, ^65^, ^66^ ^]^ Table S4, Supporting Information presents a comparison of previous research examining the rollable form factor. While there have been various attempts to increase the rolling radius, the present work is the first study of energy absorption capacity using a porous structure in a rollable form factor integrated with multifunctional applications. The rollable self‐healing touchpad with the properties listed above was fabricated by rolling the 8 cm × 5 cm electrode film on a 8 mm axis. The film was rolled up and stretched out repeatedly (Figure 6f). When the film was cut in half and healed under 60 °C for 1.5 h, the touchpad worked perfectly, showing performance similar to that of the original touch pad (Figure 6g and Video S4, Supporting Information). The resilience of PVB‐COO‐Zn was evaluated through the cyclic tensile test. As a result, the hysteresis loop was observed, indicating that the Zn‐carboxylate coordination bond served as a sacrificial bond that could effectively dissipate energy (Figure S24, Supporting Information).^[^ ^67^ ^]^ The rollable self‐healing touchpad demonstrated herein has potential for practical use in applications requiring a rollable form factor to deliver a convenient user experience.

## 3 Conclusion

In conclusion, we designed and developed Zn clusters with multifunctions (triboelectricity, shape memory, self‐healing, and optical sensing) combined with a rollable form factor for the first time. By introducing —COOH in the PVB side chain (PVB‐COOH) through the ring opening reaction and coordination with Zn ions, the ion interaction between —COO^−^and Zn^2+^ leads to the formation of Zn clusters. The Zn clusters formed pores, acted as positive tribomaterial, and created physical crosslinking points. The Zn clusters also produced a hierarchical porous structure depending on the Zn amount, which ranged from 200 to 1400 nm. As a result, porous structures are applied to corona charge injection, optical sensing for charge containers, and message transmission. A hidden tag was produced for anti‐counterfeit by utilizing pores, which is distinctive from the current practical applications for this type of material. In TENG, the output performance was increased as the surface charge increased due to the Zn doping effect. The maximum output performance (144 V, 12 µA) was generated at PVB‐COO‐Zn 20. Furthermore, the output performance was enhanced by corona charge injection under porous structures. Injecting positive charge into PVB‐COO‐Zn 20 increased the output performance (248 V) to 24 times higher than those of non‐porous structures (10 V). Further, the reversibility of physical crosslinks by the ionic interaction in Zn clusters endowed shape memory and self‐healing, which was combined with rollability. Repetitive rollability was enhanced by introducing a long alkyl chain and physical crosslinking by Zn clusters. The stability of repetitive rolling up and out was retained from the Zn clusters (PVB‐COO‐Zn), as it was shown to be 13 times more stable than when there is no long alkyl chain (PVB). Furthermore, the porous structure dissipated the stress and imparted damage resistance to the form factor through energy absorption. This study provides a new strategy for multifunctional applications (shape memory rollable‐TENG, rollable self‐healing touchpad, and hidden tag) integrated with a rollable form factor to meet the demand for devices that can be used in complex situations and physical arrangements.

## 4 Experimental Section

### Materials

PVB (with acetal content 63 mol%, hydroxyl content 36 mol%, acetate content 3 mol%) (*M*_w_ = 19 000, grade: BL‐1) was purchased from Sekisui Chemical Co. Ltd., Japan. Zinc chloride (98%), DDSA (90%, mixture of isomers), and triethylamine were purchased from Sigma‐Aldrich. *N*,*N*‐dimethylformamide (DMF, 99.5%) was purchased from Ducksan, Korea. Carbon black (Super P Conductive Carbon Black) was purchased from MTI KOREA.

### Fabrication of PVB‐COO‐Zn

To fabricate PVB‐COO‐Zn, PVB‐COOH was prepared first: To begin, 8 g of PVB was dissolved in 40 mL of DMF. Next, 0.04 g (0.125 mmol), 0.08 g (0.25 mmol), and 0.16 g (0.5 mmol) of DDSA were, respectively, added to initiate the ring opening of —OH in PVB with DDSA. Simultaneously, as catalyst, 4‐dimethylaminopyridine (DMAP) was added, and the corresponding mass ratio of DDSA to DMAP was 10:1. The mixture was then heated at 60 °C for 1 h and 90 °C for 9 h. Following the completion of the ring opening reaction, the mixture was cooled to room temperature and precipitated in a solution of 10 mL methanol and 1000 mL pure water. The PVB‐COOH was then vacuum dried for 24 h at 30 °C in oven. In a typical fabrication process, PVB‐COO‐Zn polymers were prepared by reacting PVB‐COOH with the addition of ZnCl_2_ solution. Next, 0. 5 g of PVB‐COOH was dissolved in ethanol (EtOH) (1.7 mL). After solvation, ZnCl_2_ solution (0.5 mL, 110 mg mL^−1^) was added dropwise. Then, Et_3_N (0.3 mL) was added into the solution by dropping. Finally, the mixture was stirred for 12 h at room temperature.

### Characterization and Measurements

For detailed explanations, refer to the Supporting Information.

### DFT Calculations

DFT calculations were conducted using the ORCA 4.0.1.2 program supported by Avogadro software.^[^ ^68^ ^]^ The BP86 functional with the def2‐SVP and def2/J basis sets were used to optimize the model geometries. The binding energy (∆*E*_b_) value was calculated as the energy differences between the final structure and its individual components as follows:

(3) $$\Delta E_{b}=E_{\mathrm{PVBCOOZN}}-\left(E_{\mathrm{PVBCOO}}^{\;-}+E_{\mathrm{Zn}}\right)$$

where *E*_PVBCOOZN_, $E_{\mathrm{PVBCOO}}^{\;-}$, and *E*_Zn_ are the absolute energies obtained for the PVBCOOZN, PVBCOO^−^, and Zn models following the geometry optimization.

### Fabrication of Hidden Tag

To fabricate the hidden tag, a hidden layer and a character layer were prepared. To make the hidden layer, 0.4 mL of EtOH was added to 0.3 g of PVB‐COO‐Zn x solution and mixed with 6 mg of carbon black at 30 °C for 24 h. After mixing, the solutions were casted on polyester (PET) film in 2 cm × 6 cm size and dried. To make the character layer, dye solution was made by mixing 0.5 g of Blue type 35 dye and Blue type 70 dye with PVB solution (PVB 0.5 g, EtOH 1 g) for the words “FAKE” and “REAL,” respectively.

### Fabrication of Shape Memory Rotating‐TENG

To fabricate SMR‐TENG, Cu tape (1 cm × 4.5 cm, thickness: 100 µm) was attached to the inside of the stator, and PTFE film (thickness: 50 µm) was attached to the whole inside of the stator. Finally, PVB‐COO‐Zn 15 film (1 cm × 2 cm, thickness: 74 µm) was attached on the blade.

### Fabrication of Rollable Self‐Healing Touchpad

To fabricate the rollable self‐healing touchpad, 1 g solution of PVB‐COO‐Zn 10 fabricated as described above was mixed with 15 wt% of carbon black and the addition of 1 mL EtOH. After stirring for 24 h at 40 °C, the solution was casted and dried at 30 °C for 12 h and 60 °C for 1 h.

## Conflict of Interest

The authors declare no conflict of interest.

## Supporting information

Supporting Information

Supplemental Video 1

Supplemental Video 2

Supplemental Video 3

Supplemental Video 4

## Data Availability

Research data are not shared.
