# Supplementary material for: Controllable Physical Synergized Triboelectricity, Shape Memory, Self‐Healing, and Optical Sensing with Rollable Form Factor by Zn cluster
Source: Adv Sci (Weinh). 2022 Apr 22;9(18):2200441. doi: 10.1002/advs.202200441 (PMC9366568; doi:10.1002/advs.202200441)
Supplement: Supplementary file 1 — Supporting Information [file ADVS-9-2200441-s005.pdf]

ADVANCED SCIENCE
Open Access

Supporting Information

for *Adv. Sci.*, DOI 10.1002/advs.202200441

Controllable Physical Synergized Triboelectricity, Shape Memory, Self-Healing, and Optical Sensing with Rollable Form Factor by Zn cluster

*Dahye Ahn, Jingzhe Sun, Seunghye Han, Jiwoo Lee, Songah Jeong, Seokjun Cha, Seonmyeong Noh, Hyeongsub Choi, Bingqi Ren, Hyeonseok Yoon, Hyungwoo Kim and Jong-Jin Park**

Supporting Information

Controllable Physical Synergized Triboelectricity, Shape Memory, Self-Healing, and Optical Property with Rollable Form Factor by Zn cluster

*Dahye Ahn, Jingzhe Sun, Seunghye Han, Jiwoo Lee, Songah Jeong, Seokjun Cha, Seonmyeong Noh, Hyeongsub Choi, Bingqi Ren, Hyeonseok Yoon, Hyungwoo Kim, Jong-Jin Park**

Department of Polymer Science and Engineering, Chonnam National University, Gwangju 61186, Republic of Korea
E-mail: jjpark@chonnam.ac.kr

Experimental Section

Characterization and measurements The thermomechanical property and stress-relaxation behavior were measured by DMA (DMA2980, TA, USA). For cyclic thermomechanical experiment, the sample was first stretched by ε_p at 40 ℃. Then, while keeping the strain held constant, the temperature was gradually decreased to 25 ℃. The sample was held isothermally for 2 min after reaching 25 ℃. By suddenly removing the external force, the temporary fixed strain ε_u was achieved. The free recovery was activated by gradually increasing the temperature to the recovery temperature (40 ℃) and the recovery strain (ε_r) was measured. Thereafter, the ratio of shape fixity ($R_f = \varepsilon_u / \varepsilon_p$) and shape recovery ($R_r = (\varepsilon_u - \varepsilon_r) / \varepsilon_u$) was calculated.

Experimental Section

Characterization and measurement

Material characterization : Fourier-transform infrared spectroscopy (FT-IR, SHIMADZU, IRAffinity-1S) was used from 4000 to 500 cm^{-1} by 32 scans with a resolution of $4cm^{-1}$. ^{1}H NMR spectra were recorded by MestReNova 500 MHz NMR spectrometers at 25 °C. Differential scanning calorimetry (DSC) was performed using a Mettler Toledo DSC 3 instrument under N_2 atmosphere with the heating rate of 10 K min^{-1}. Thermogravimetric analysis (TGA) was recorded using an Mettler Toledo TGA 2 instrument under N_2 atmosphere with the heating rate of 10 K min^{-1}. UV-Vis spectra were observed by an Optizen 2120 UV spectrophotometer with a plastic cuvette with a 10-mm path length. High-performance x-ray photoelectron spectroscopy (HP-XPS, K-ALPHA +) was used to analyze the chemical composition of the PVB-COOH and PVB-COO-Zn.

Surface morphology analysis: The morphology of porous structures in PVB-COO-Zn were observed using Field emission scanning electron microscopy (FE-SEM, JSM-7900F, JEOL) and atomic force microscopy (AFM, XE-100).

Measurement of triboelectric output performance: For measuring triboelectric output performance of PVB-COO-Zn, PVB-COO-Zn solutions according to $ZnCl_2$ amount were prepared and casted on glass substrate using doctor blade (70 μm) in the size of 4 cm × 4 cm. The casted films were dried in 30 °C for 24 h. The triboelectric output performance in contact-separation mode was conducted with a self-made compress/release machine under an external force of 80 N and a frequency of 2.5 Hz. Output performance of TENG were measured using a DPO2021B digital phosphor osiloscope and a RIGOL DIM3058E digital multimeter. The open-circuit voltage was obtained with the oscilloscope with an internal resistance of 1 MΩ. Surface charge density was obtained by Kiethley 6514 system electrometer. For measuring the wind speed, wind-speed tester (Testo, Testo 416) was used in the front center of the SMR-TENG to precisely measure the wind speed.

Stability test of repetitive rolling: The stability test when rolling up and out was carried out on the wrapped PVB-COO-Zn carbon electrode film around a supporter with a diameter of 8 mm. The resistance was measured by 4-point probe of Keithley 2450 sourcemeter.

Dynamic Mechanical Analysis : Thermomechanical property and stress-relaxation behavior were measured by dynamic mechanical analysis (DMA2980, TA, USA). For cyclic thermomechanical experiment, the sample was first stretched by ε_p at 40 ℃. Then, while keeping the strain held constant, the temperature was gradually decreased to 25 ℃. The sample was held isothermally for 2 min after reaching 25 ℃. By suddenly removing the external force, the temporary fixed strain ε_u was achieved. The free recovery was activated by gradually increasing the temperature to the recovery temperature (40 ℃) and the recovery strain (ε_r) was measured. Thereafter, the ratio of shape fixity ($R_f = \varepsilon_u / \varepsilon_p$) and shape recovery ($R_r = (\varepsilon_u - \varepsilon_r) / \varepsilon_u$) was calculated. For stress-relaxation experiment, the samples according to Zn amount were stretched to 1% of strain for 30 min. In all DMA analysis, the sample size was 5 mm (W) × 15 mm (L) × 0.8 mm (T).

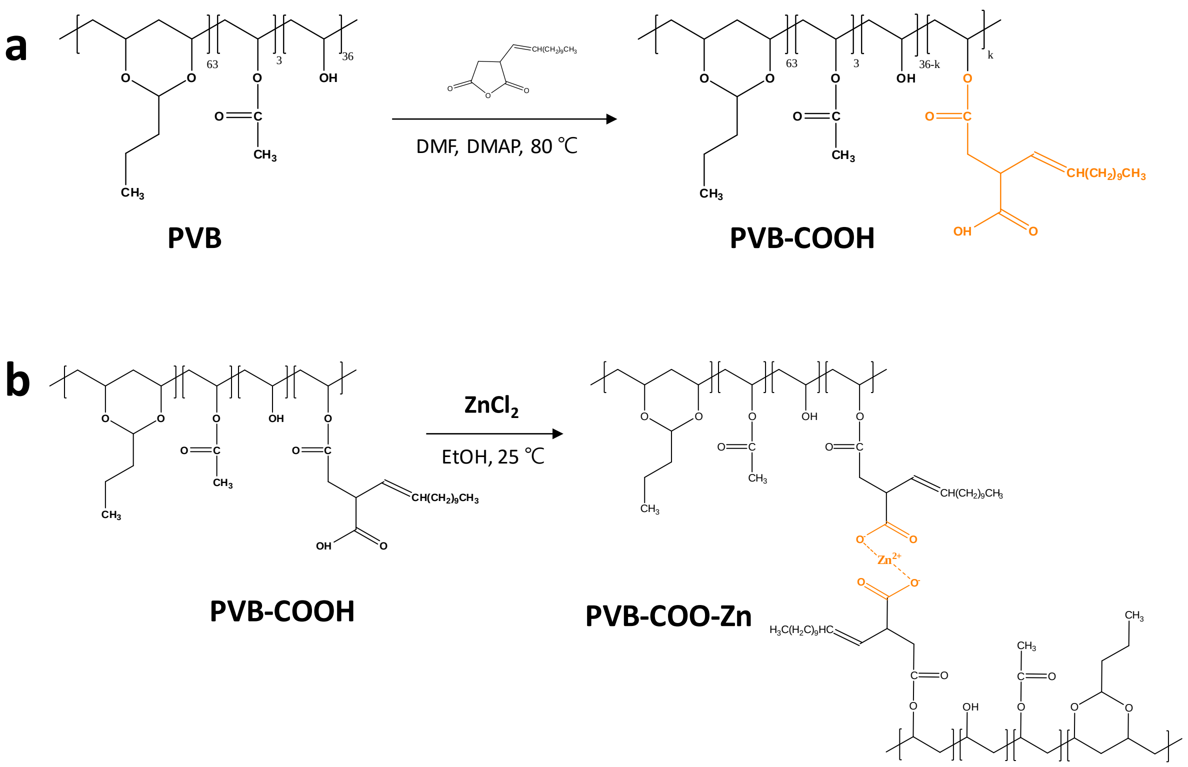

Figure S1. Modification of Poly (vinyl butyral) using Dodecenylsuccinic anhydride

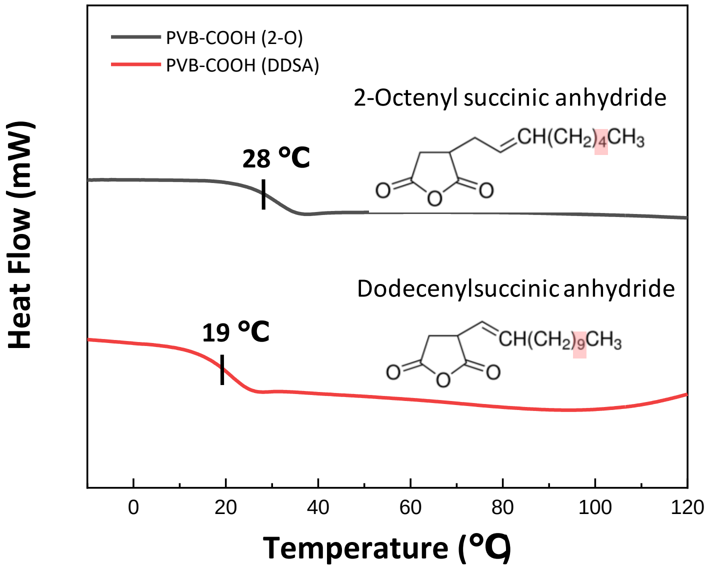

Figure S2. Comparison of T_g when using 2-Octenyl succinic anhydride (2-O) and Dodecenylsuccinic anhydride (DDSA) for PVB modification.

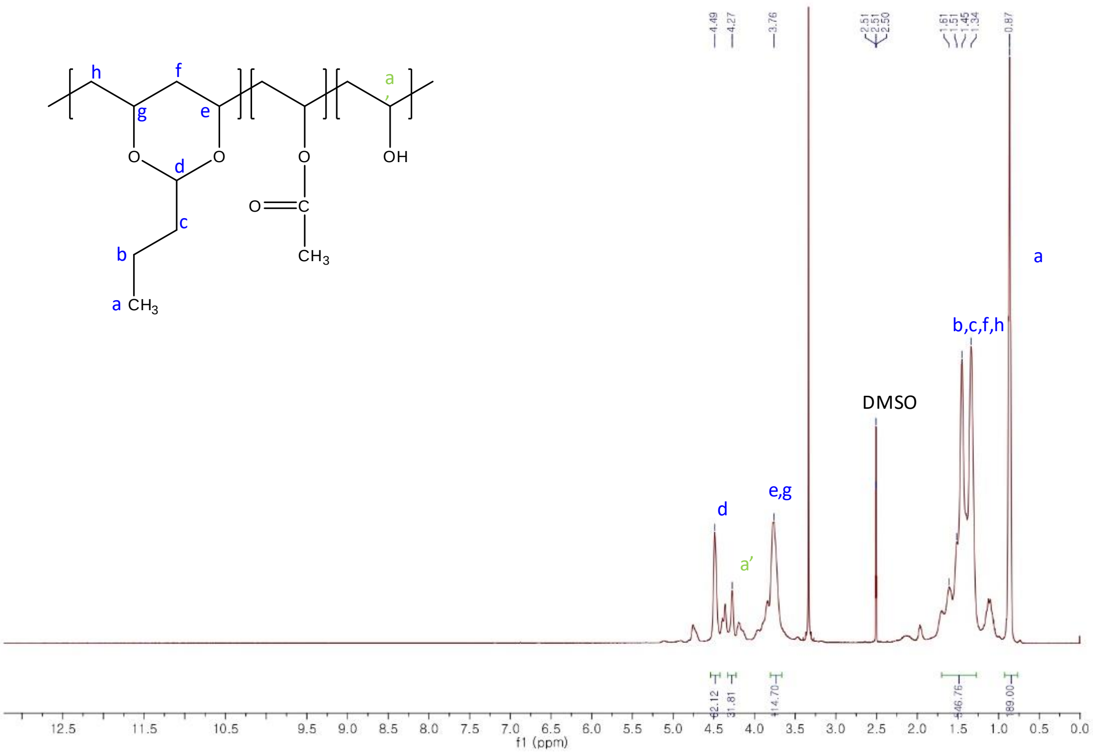

Figure S3. ^{1}H NMR data of PVB

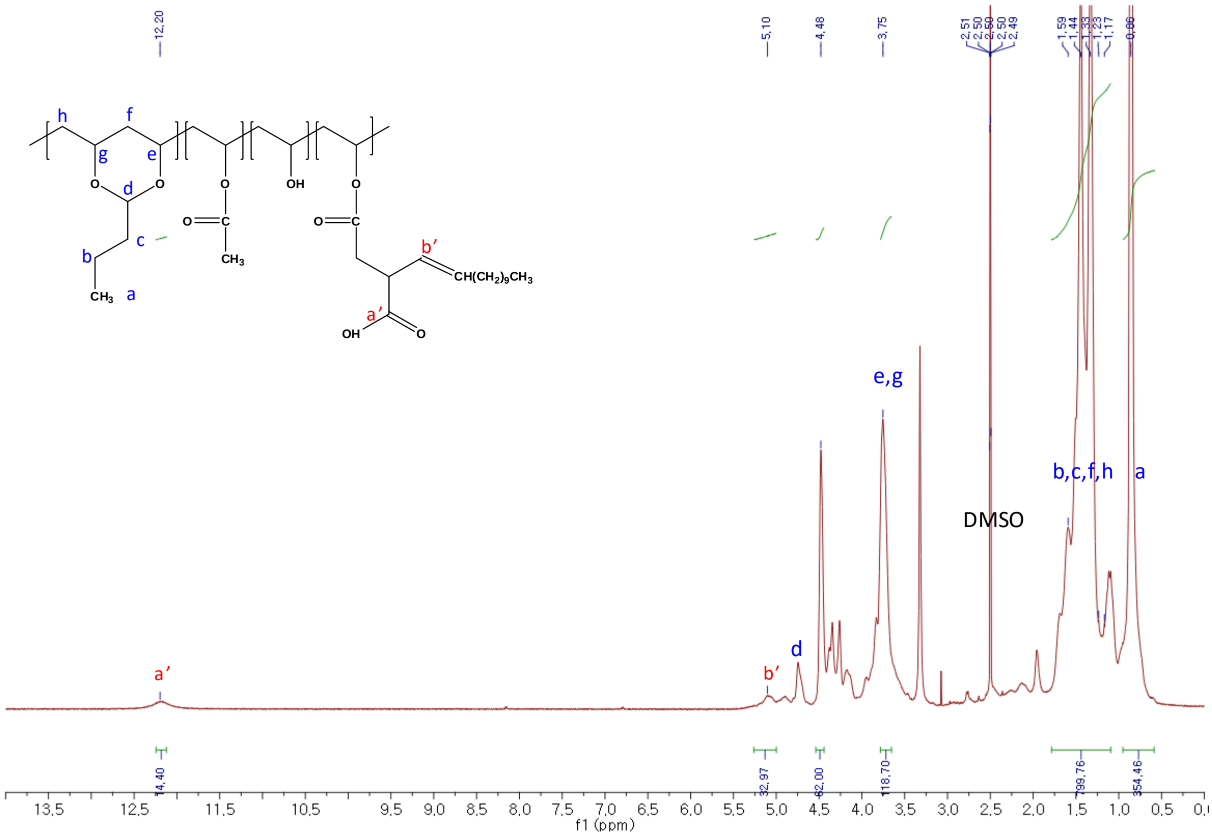

Figure S4. ^{1}H NMR data of PVB-COOH

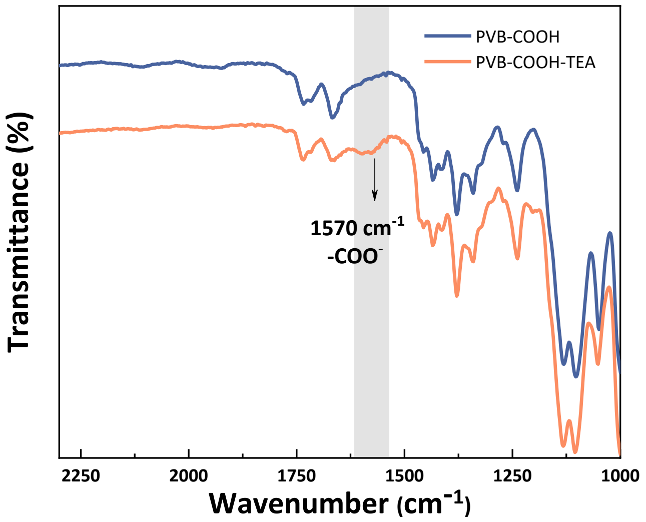

Figure S5. FT-IR of PVB-COOH and PVB-COOH-TEA

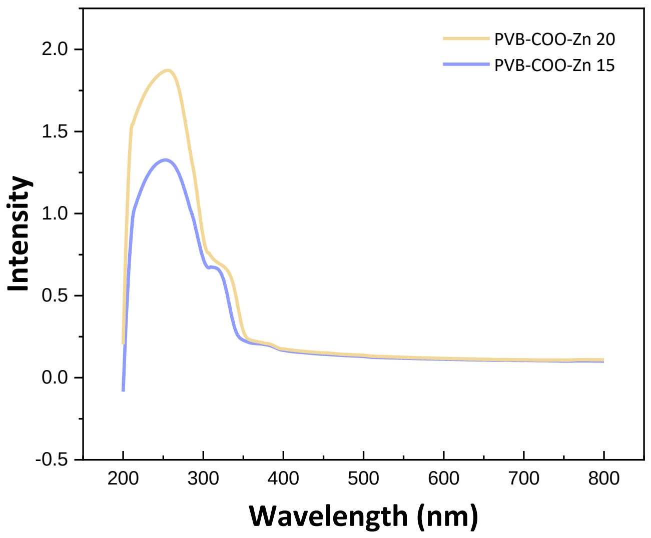

Figure S6. UV-vis-spectra of PVB-COO-Zn samples with different amount of Zn.

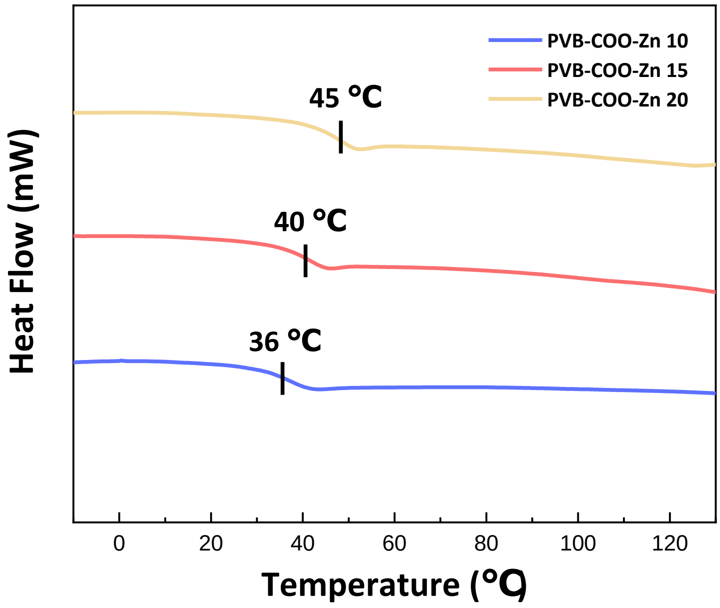

Figure S7. DSC graph of PVB-COO-Zn according to Zn amount.

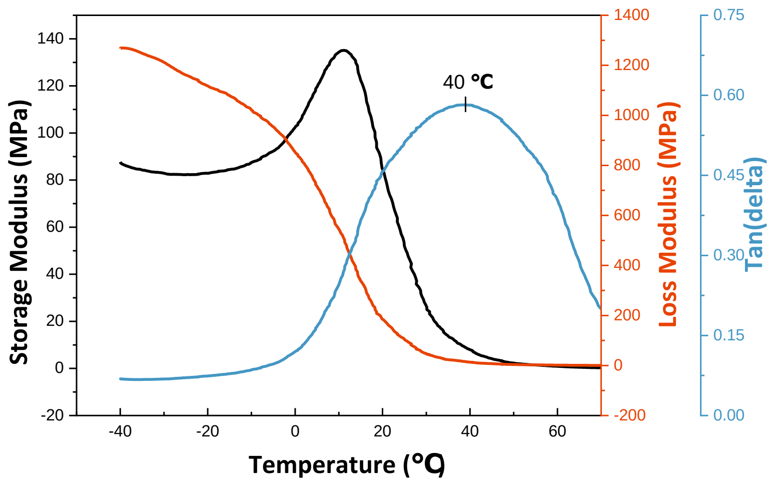

Figure S8. Storage modulus, loss modulus, and tan δ obtained by DMA

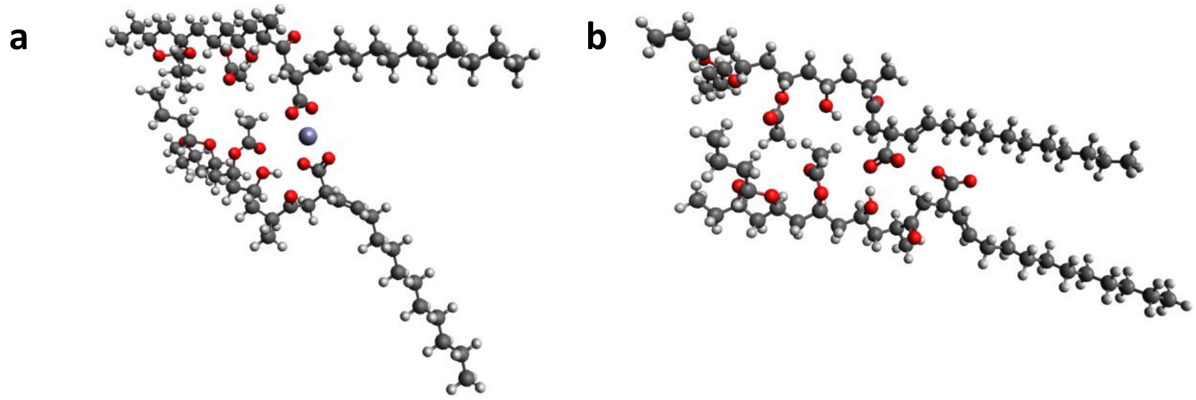

Figure S9. Optimized geometrical structures for the models obtained using DFT calculations. (a) PVB-COO-Zn (b) PVB-COO^-

Density functional theory (DFT) calculations were performed for miniaturized models to examine the interaction between PVB-COOH and Zn. Two PVB-COO^- segments were employed as miniature models for the polymer and the binding ability of a Zn ion with the two model segments was calculated using the BP86 functional with def2-SVP and def2/J basis sets. Before calculating the interaction, the model structures were optimized. Figure S8 presents the optimized geometries of the 000 model in the absence and presence of Zn. It was found that the Zn ion is located between the COO^- groups of the model polymers with the distance of 2.04−2.06 Å. The binding energy, $\Delta E_b = E_{\mathrm{PVBCOOZn}} - (E_{\mathrm{PVBCOOs}} + E_{\mathrm{Zn}})$ was calculated to be −0.036, indicating the binding of the PVBCOO with Zn is thermodynamically favorable (Table S1).

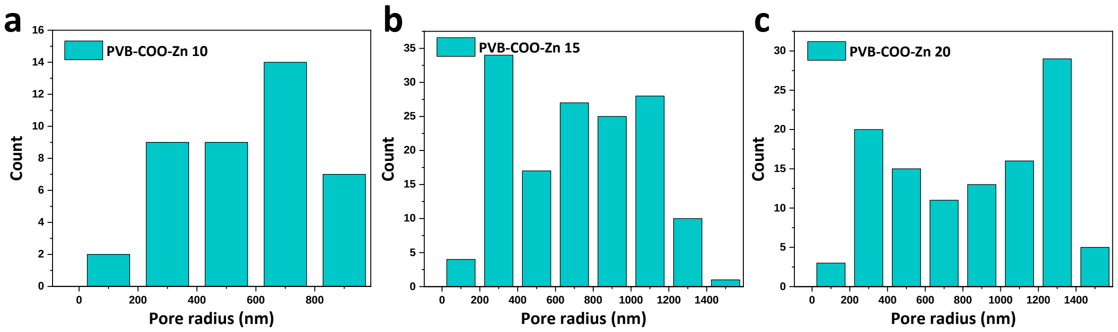

Figure S10. Pore size distribution of PVB-COO-Zn according to Zn amount calculated by using ImageJ. (a) Zn 10 wt% (b) Zn 15 wt% (c) Zn 20 wt%

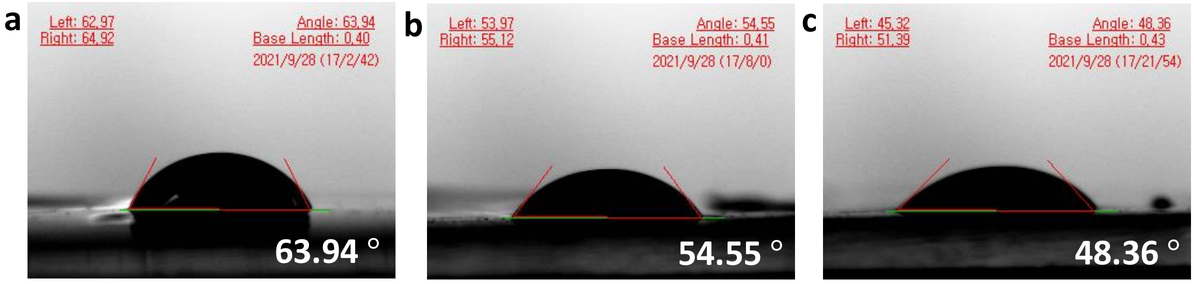

Figure S11. Pore size distribution of PVB-COO-Zn according to Zn amount (a) Zn 10 wt% (b) Zn 15 wt% (c) Zn 20 wt%

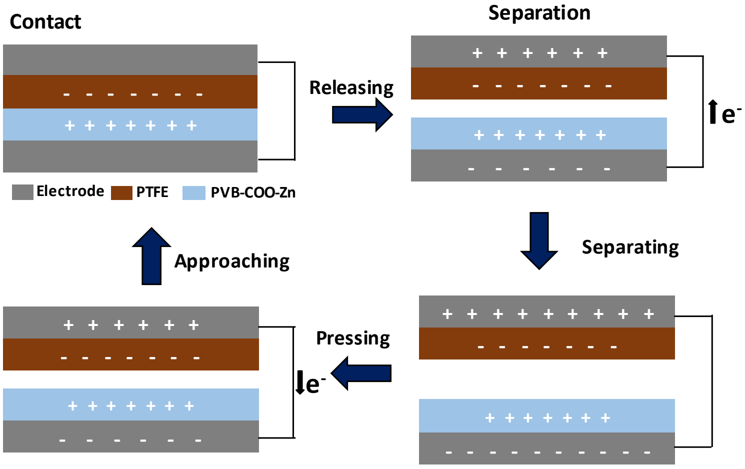

Figure S12. Working mechanism of TENG in PVB-COO-Zn system

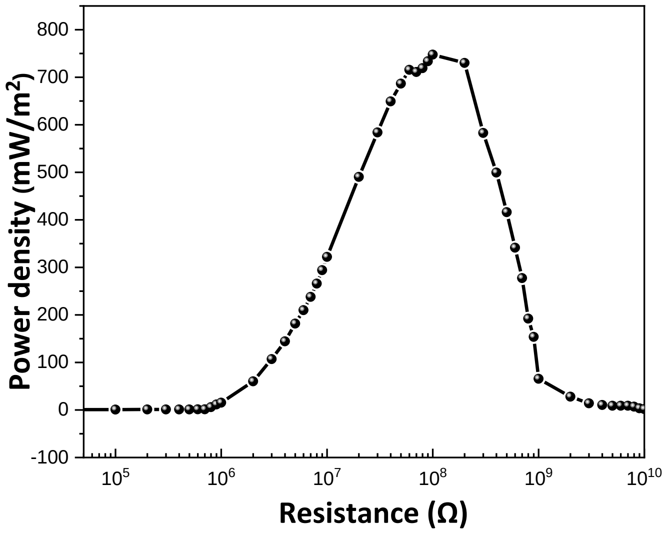

Figure S13. Power density of PVB-COO-Zn 20.

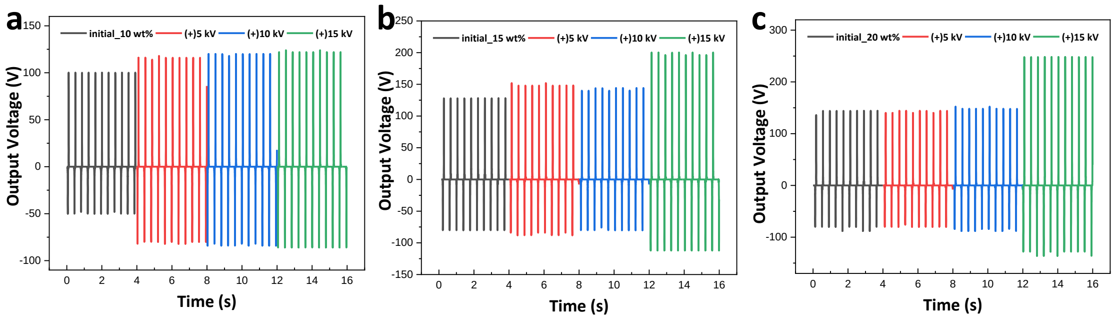

Figure S14. Positive ion charge injection according to injection voltage (0, 5, 10, 15 kV) and Zn amount. (a) Zn 10 wt% (b) Zn 15 wt% (c) Zn 20 wt%

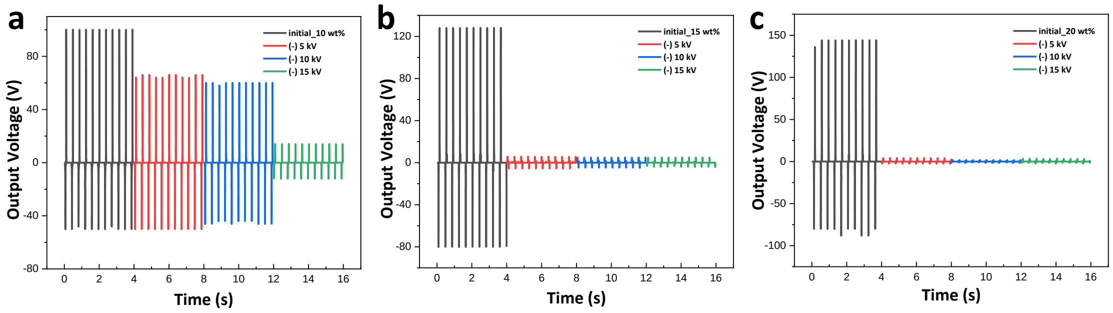

Figure S15. Negative ion charge injection according to injection voltage (0, 5, 10, 15 kV) and Zn amount. (d) Zn 10 wt% (e) Zn 15 wt% (f) Zn 20 wt%

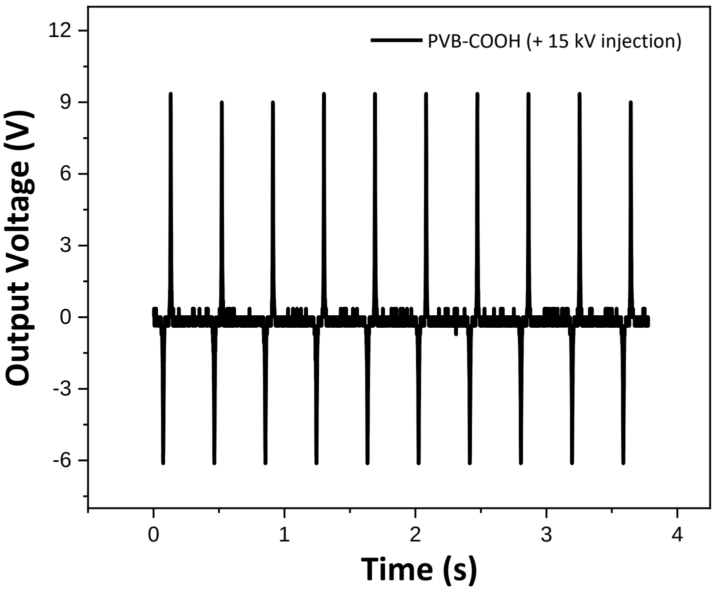

Figure S16. Output voltage of PVB-COOH after corona charge injection

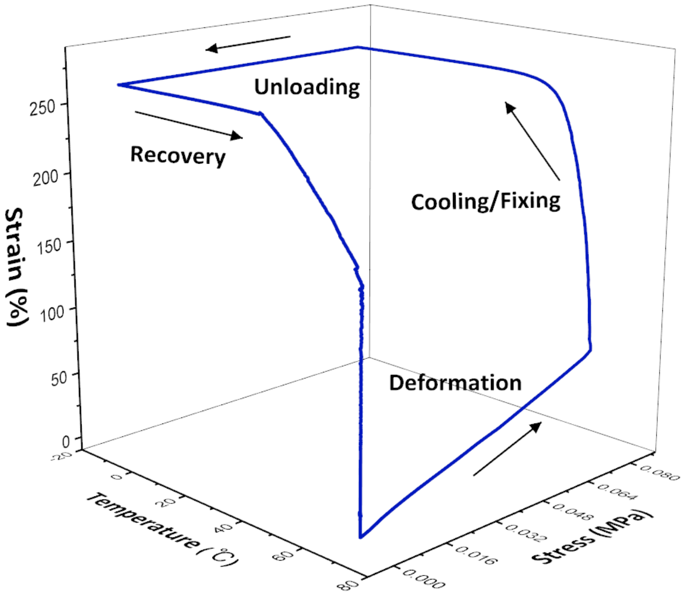

Figure S17. Three dimensional test record of cyclic thermomechanical test of PVB-COO-Zn 15

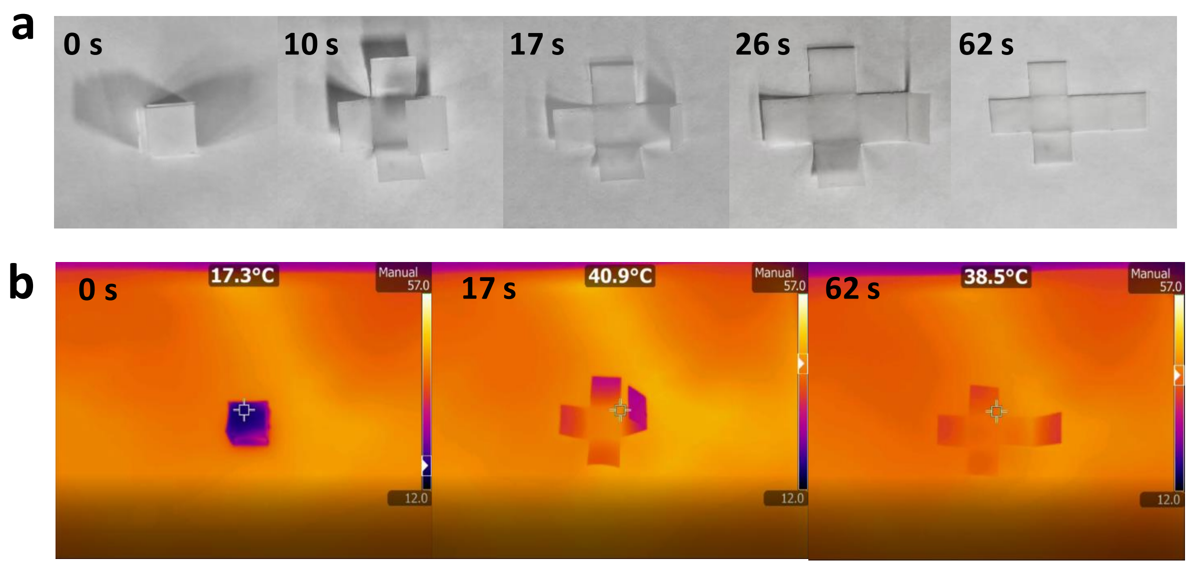

Figure S18. (a) Photographic images of shape memory properties of PVB-COO-Zn 15 in box shape. (b) IR camera images of (a) at 0 s, 17s, and 62s.

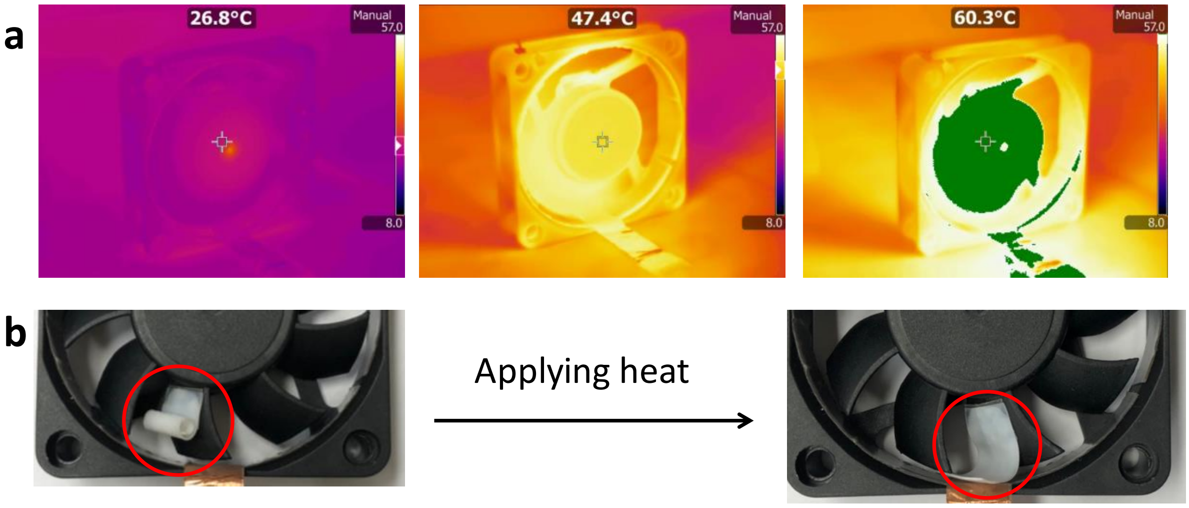

Figure S19. (a) IR camera images of SMR-TENG before and after blade rotates. (b) Photographic images of film on blade before and after changing its shape according to the temperature of blade.

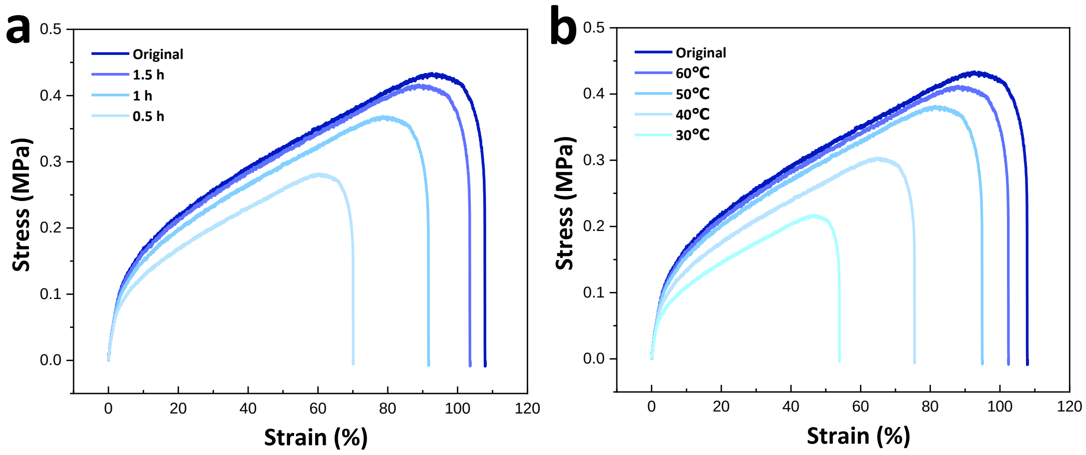

Figure S20. Stress-strain curves of the original and self-healed PVB-COO-Zn (a) under different healing time and (b) different healing temperature.

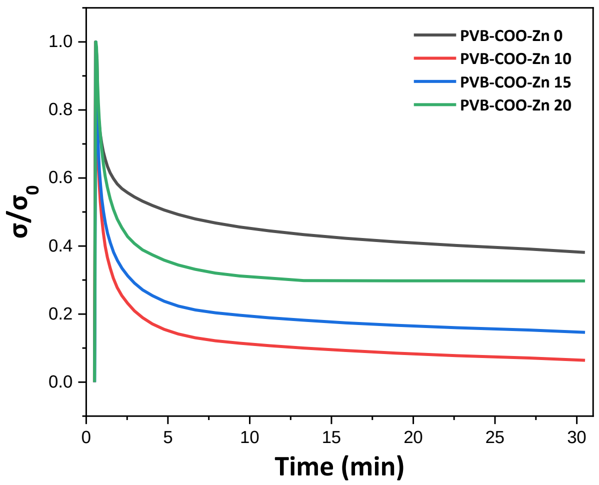

Figure S21. Stress-relaxation of PVB-COO-Zn samples at 25 ℃ under 1 % of strain.

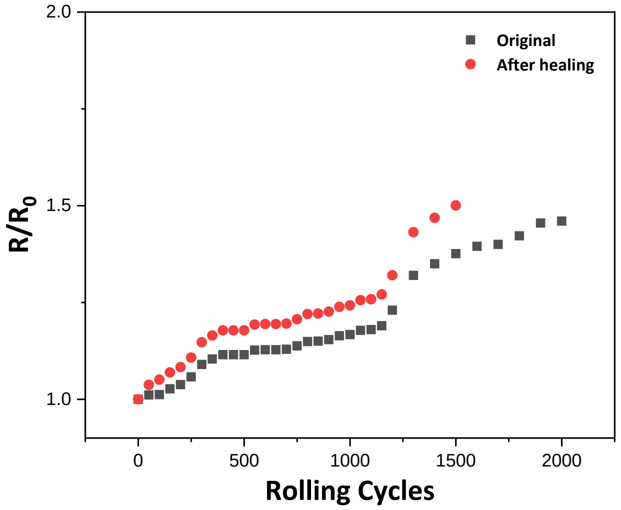

Figure S22. Rolling stability of PVB-COO-Zn after healing.

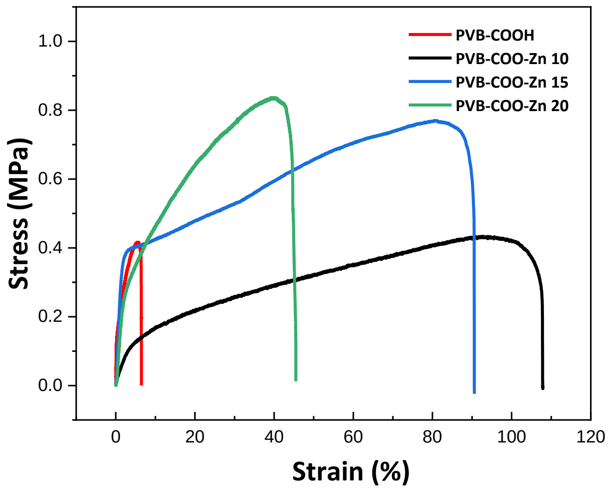

Figure S23. Stress-strain curve of PVB-COO-Zn acoording to Zn amount.

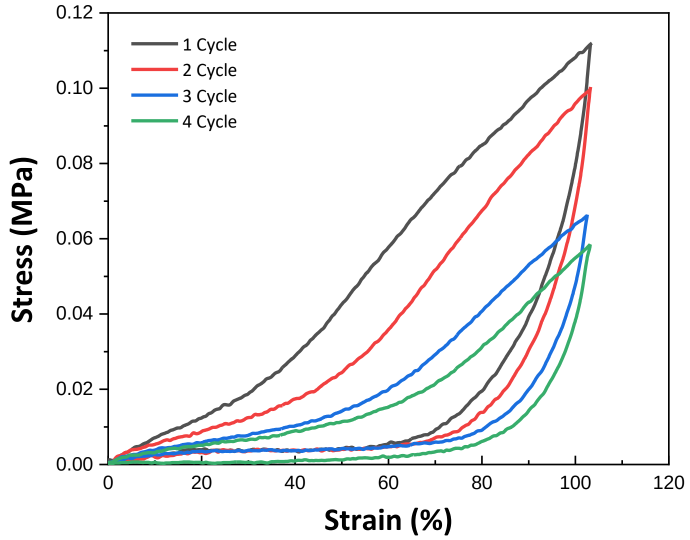

Figure S24. Successive cyclic tensile test at the strain of 100%

Table S1. Comparison table of this work and the result of Zn coordination in previous studies

Reference	Material (ligand/metal)	Application
[1] 2012 *J. Mater. Chem*	Carboxylate /Zinc acrylate ($Zn(AA)_2$)	Light-emitting diode(LEDs)
[2] 2016 *J. Am. Chem. Soc*	Pyridyl nitrogen /Zinc chloride ($ZnCl_2$)	Stretchable self-healing polymeric dielectrics
[3] 2018 *Adv. Funct. Mater*	Histidine /ZnCl-decorated epoxidized natural rubber (ENR-Zn)	Self-heaing sensor with tunable positive/negative piezoresistivity
[4] 2018 *Nat. Commun*	Carboxylate /Zinc chloride ($ZnCl_2$)	Orthopedic immobilization Conductive composite/adhesives 3D printing
[5] 2019 *Sci. Adv*	Carboxylate /Zinc palmitate(ZnPa)	-
[6] 2020 *RSC. Adv*	Carboxylate /Zinc oxide (ZnO)	-

[7] 2021 *Angew. Chem. Int*	H_2O /Zinc chloride ($ZnCl_2$)	Flexible zinc-ion hybrid capacitor
[8] 2021 *ChemistySelect*	Imidazole /Zinc (Zn)	Organic light-emitting diodes (OLEDs)<br>Anti-counterfeiting<br>Latent finger print detection (LFPs)
[9] 2021 *Green. Chem*	Hydroxyl group /Zinc chloride ($ZnCl_2$) and Aluminium chloride ($AlCl_3$)	Wearable monitors based on strain-sensitivity conductivity
[10] 2022 *Adv. Funct. Mater*	H_2O and SO_4^{2-} /Zinc sulfate ($ZnSO_4$)	Dendrite-free zinc battery
**This work**	**Carboxylate/$ZnCl_2$**	**Hidden tag**<br>**Shape memory rotating TENG**<br>**Rollable self-healing touchpad**

Table S2. Glass transition temperature of PVB-COOH according to DDSA amount.

**Material**	**Molar ratio (-OH in PVB : DDSA)**	**T_g (°C)**	**Triboelectric output voltage at Zn 10 wt% (V)**
	1:1	29	85
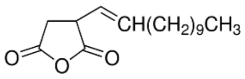	1:2	26	80
Dodecenylsuccinic anhydride (DDSA)	1:4	19	100
	1:8	16	50

As the amount of anhydride increased, the glass transition temperature decreased, which facilitates rollability, but the triboelectric output voltage at 10 wt% of Zn was rather decreased from molar ratio 1:8. Considering two factors, molar ratio of 1:4 appeared to be the most suitable.

Table S3. Main parameters calculated for the binding modes with PVB-COOs and Zn.

E_{PVBCOOZn} (a.u.)	E_{PVBCOO^-} (a.u.)	E_{Zn} (a.u.)	ΔE_{b} (a.u.)
−5642.615	−3863.190	−1779.389	−0.036

Table S4. Previous papers related with fully rollable form factors

**Reference**	**Material**	**Rolling diameter**	**Rolling cycles for stability test**	**Roll numbers**	**Application**
[11] 2009 *Adv. Mater*	4‘ Ni-coated SiO2/Si wafer	8 mm	Several cycles	1	Transparent electrode in piezoelectric energy harvesting nanodevices
[12] 2015 *ACS Nano*	Paper	None	None	1	Triboelectric nanogerator for self-powered sound recording
[13] 2018 *ACS Nano*	Poly (vinylidene fluoride)-Niobate-based Nanogenerator	8 mm	200	Not mentioned	Piezoelectric nanogenerator

[14] 2019 *Adv. Mater*	Graphene oxide film	Not mentioned	None	1	Hygroelectric nanogenerator
[15] 2019 *NPG Asia Materials*	Silicone rubber	< 1mm	None	-	Strain/temperature sensor
[16] 2020 *ACS AMI*	Porous PVDF-co-HFP intertwined nanosponge	4 cm	5,000	Not mentioned	Lithium-ion batteries
[17] 2021 *Nanoscale Advances*	MoS_2/TOCN composite material	5 mm	100	4	Photodetector
**This work**	**PVB-COO-Zn**	**≤8 mm**	**2,000**	**7**	**Hidden tag**<br>**Shape memory rotating TENG**<br>**Rollable self-healing touchpad**

References

[1] J. Wang, H. X. Shen, C. F. Wang, S. Chen, *J. Mater. Chem.* **2012**, *22*, 4089.

[2] Y. L. Rao, A. Chortos, R. Pfattner, F. Lissel, Y. C. Chiu, V. Feig, J. Xu, T. Kurosawa, X. Gu, C. Wang, M. He, J. W. Chung, Z. Bao, *J. Am. Chem. Soc.* **2016**, *138*, 6020.

[3] X. Liu, G. Su, Q. Guo, C. Lu, T. Zhou, C. Zhou, X. Zhang, *Adv. Funct. Mater.* **2018**, *28*, 1.

[4] J. C. Lai, L. Li, D. P. Wang, M. H. Zhang, S. R. Mo, X. Wang, K. Y. Zeng, C. H. Li, Q. Jiang, X. Z. You, J. L. Zuo, *Nat. Commun.* **2018**, *9*, DOI 10.1038/s41467-018-05285-3.

[5] J. J. Hermans, L. Baij, M. Koenis, K. Keune, P. D. Iedema, S. Woutersen, *Sci. Adv.* **2019**, *5*, 1.

[6] P. Junkong, R. Morimoto, K. Miyaji, A. Tohsan, Y. Sakaki, Y. Ikeda, *RSC Adv.* **2020**, *10*, 4772.

[7] C. Wang, Z. Pei, Q. Meng, C. Zhang, X. Sui, Z. Yuan, S. Wang, Y. Chen, *Angew. Chemie - Int. Ed.* **2021**, *60*, 990.

[8] R. M. Kempegowda, M. K. Malavalli, G. H. Malimath, L. Naik, K. B. Manjappa, *ChemistrySelect* **2021**, *6*, 3033.

[9] Y. Xi, L. Zhang, Y. Tian, J. Song, J. Ma, Z. Wang, *Green Chem.* **2022**, *24*, 885.

[10] Z. Miao, F. Zhang, H. Zhao, M. Du, H. Li, H. Jiang, W. Li, Y. Sang, H. Liu, S. Wang, *Adv. Funct. Mater.* **2022**, *2111635*, 1.

[11] D. Choi, M. Y. Choi, W. M. Choi, H. J. Shin, H. K. Park, J. K. Seo, J. Park, S. M. Yoon, S. Chae, Y. H. Lee, S. W. Kim, J. Y. Choi, S. Y. Lee, J. M. Kim, *Adv. Mater.* **2010**, *22*, 2187.

[12] Z. L. Xing Fan, Jun Chen, Jin Yang, Peng Bai, Z. L. Wang, *ACS Nano* **2015**, *9*, 4236.

[13] C. Zhang, Y. Fan, H. Li, Y. Li, L. Zhang, S. Cao, S. Kuang, Y. Zhao, A. Chen, G. Zhu, Z. L. Wang, *ACS Nano* **2018**, *12*, 4803.

[14] C. Yang, Y. Huang, H. Cheng, L. Jiang, L. Qu, *Adv. Mater.* **2019**, *31*, 1.

[15] G. Lee, Y. W. Choi, T. Lee, K. S. Lim, J. Shin, T. Kim, H. K. Kim, B. K. Koo, H. B. Kim, J. G. Lee, K. Ahn, E. Lee, M. S. Lee, J. Jeon, H. S. Yang, P. Won, S. Mo, N. Kim, M. H. Jeong, Y. Roh, S. Han, J. S. Koh, S. M. Kim, D. Kang, M. Choi, *NPG Asia Mater.* **2019**, *11*, 67

[16] S. Oh, V. H. Nguyen, V. T. Bui, S. Nam, M. Mahato, I. K. Oh, *ACS Appl. Mater. Interfaces* **2020**, *12*, 11657.

[17] C. Yoo, T. J. Ko, S. S. Han, M. S. Shawkat, K. H. Oh, B. K. Kim, H. S. Chung, Y. Jung, *Nanoscale Adv.* **2021**, *3*, 3028.
